# High hospital readmission rates for patients aged ≥65 years associated with low socioeconomic status in a Swedish region: a cross-sectional study in primary care

**DOI:** 10.1080/02813432.2018.1499584

**Published:** 2018-08-23

**Authors:** Jacques Shebehe, Anders Hansson

**Affiliations:** a The University Healthcare Research Centre, Faculty of Medicine and Health, Örebro University, Örebro, Sweden;; b Academy of Sahlgrenska, Institute of Medicine, University of Gothenburg, Gothenburg, Sweden

**Keywords:** Aged, patient readmissions, quality of health care, primary health care, socioeconomic factors

## Abstract

**Objective:** There is a presumption that hospital readmission rates amongst persons aged ≥65 years are mainly dependent on the quality of care. In this study, our primary aim was to explore the association between 30-day hospital readmission for patients aged ≥65 years and socioeconomic characteristics of the studied population. A secondary aim was to explore the association between self-reported lack of strategies for working with older patients at primary health care centres and early readmission.

**Design:** A cross-sectional ecological study and an online questionnaire sent to the heads of the primary health care centres. We performed correlation and regression analyses.

**Setting and subjects:** Register data of 283,063 patients in 29 primary health care centres in the Region Örebro County (Sweden) in 2014.

**Main outcome measure:** Thirty-day hospital readmission rates for patients aged ≥65 years. Covariates were socioeconomic characteristics among patients registered at the primary health care centre and eldercare workload.

**Results:** Early hospital readmission was found to be associated with low socioeconomic status of the studied population: proportion foreign-born (*r* = 0.74; *p* < 0.001), proportion unemployed (*r* = 0.73; *p* < 0.001), Care Need Index (*r* = 0.74; *p* < 0.001), sick leave rate (*r* = 0.51; *p* < 0.01) and average income (*r* = −0.40; *p* = 0.03). The proportion of unemployed alone could explain up to 71.4% of the variability in hospital readmission (*p* < 0.001). Primary health care centres reporting lack of strategies to prevent readmissions in older patients did not have higher hospital readmission rates than those reporting they had such strategies.

**Conclusion:** Primary health care centres localized in neighbourhoods with low socioeconomic status had higher rates of hospital readmission for patients aged ≥65. Interventions aimed at reducing hospital readmissions for older patients should also consider socioeconomic disparities.

**Key Points**
- In Sweden, hospital readmission within 30 days among patients aged ≥65 has been used as a measure of quality of primary care for the elderly.
- However, in our study, elderly 30-day readmission was associated with low neighbourhood socioeconomic status.
- A simple survey in one Swedish region showed that the primary health care centres that lacked active strategies for working with aged patients did not have higher hospital readmission rates than those that reported having strategies.
- Interventions aimed at reducing elderly hospital readmissions should therefore also consider the socioeconomic disparities in the elderly.

## Introduction

The proportion of people aged over 65 years has increased in Europe and is expected to almost double over the next four decades, representing 27% of the population by 2050 [1]. Changes in lifestyle, environmental factors and medical advances mean that, nowadays, older adults can live longer with previously untreatable illnesses [2]. The growing proportion of older people with chronic illness calls for increased quality and efficiency in healthcare.

In Sweden, on average 19% (260,000 of a total of 1.35 million hospitalisations) of all hospitalisations are hospital readmissions occurring within 30 days after discharge. These have been estimated to cost SEK 2.3 billion (USD 114 million) per year [3]. Apart from the cost, being readmitted is associated with increased mortality and morbidity risk. Older patients have an increased risk of being readmitted, and they are much more negatively affected by unplanned emergency readmissions [4]. Hospital readmission is therefore being widely used as an indicator of the quality of care [1]. The Swedish government made available financial compensation and special funds for Swedish local authorities and regions to reduce avoidable hospitalisation and hospital readmission ≤30 days after discharge for patients aged ≥65 [5].

According to international literature, multi-morbidity, marital status, ethnicity and low socioeconomic status are proposed as common risk factors for hospital readmissions for older adults [6–8]. Further, rural areas, more GPs per capita and more nursing homes per capita were found to be associated with lower readmission risk, whereas more specialists per capita and hospital beds per capita were correlated with higher readmission risk [9]. Despite the increasing evidence supporting multi-factorial causes of hospital readmission for older patients, Swedish reports seem to assume deficiencies in the health-care work with older persons to be the main factor responsible for hospital readmission [10–12]. The Swedish Association of Local Authorities and Regions (SALAR), for example, reported that hospital readmission rates for patients ≥65 years could not be explained by socioeconomic factors, multi-morbidity or distance to nearest hospital [11].

In accordance with these assumptions, the results of an investigation done by Health Navigator was presented in 2014 to all primary care and health centre managers in Region Örebro County (RÖC). The report’s underlying message was that work-related deficiencies at primary health care centres (PHCCs) and local authorities could explain the differences in the number of hospital readmissions for older patients, and designated PHCCs and municipalities were considered to be able to improve their work with their elderly patients [12]. In this study, we therefore wanted to examine whether there was an association between 30-day hospital readmission for patients ≥65 years and the socioeconomic characteristics of the population in the specific PHCC areas in RÖC. We also wanted to see if there was an association between self-reported lack of strategies for working with older patients at the individual PHCC and early readmission.

## Methods

### Design

A cross-sectional ecological register case study [13] of Region Örebro County’s primary care in 2014. All 29 PHCCs in the region were included in the study. Register data and a two-item questionnaire were used.

### Outcome variable

#### Early (<30 days) hospital readmission

This study uses Swedish National Board of Health and Welfare (SNBHW) and SALAR’s definition (prior to the revision in 2014) for 30-day hospital readmissions for patients ≥65 years, irrespective of diagnosis or whether the previous incident of hospitalisation was planned or not [14]. Hospital readmission ≤30 days is calculated by dividing the number of readmission care episodes within 30 days of discharge by the total number of care episodes for patients aged ≥65 [14]. Hospital readmission was presented per 100 patients aged ≥65.

### Independent variables

Our independent variables are socioeconomic factors expressed as Care Need Index (CNI), sick-leave rate and average income, and eldercare workload expressed as the proportion of older patients registered at a PHCC, the proportion of patients aged ≥75 years taking more than ten medications and the number of resident care facility places the PHCC was responsible for. Further independent variables are self-reported lack of strategy and staff (nurses or medical doctors) for eldercare at the PHCC.

#### Socioeconomic factors: Care Need Index (CNI), sick-leave rate and average income

CNI is a regionally customised socioeconomic care need index used for calculating the allocation of resources to authorised healthcare facilities [15]. Sundquist et al. asked a selection of Swedish GPs to specify how seven different socioeconomic factors influenced their workload on a nine-point scale [16]. From their grading, the different variables were given relative weights, called CNI weights or points, given here in brackets. The variables are: number of people living alone aged ≥65 (6.15); number of people born outside the EU, in South and East Europe, Asia, Africa and South America (5.72); number of unemployed or economically inactive 16–64-year-olds (5.13); number of single parents with children aged ≤17 (4.19); number of people who moved into the area in the past year (4.19); number of low-educated individuals aged 25–64 (3.97); and the number of children under 5 years (3.23) [17]. The normalized CNI, calculated by dividing the CNI points of each health centre per person by the median of the CNI points for all health centres per person, was used [15]. The higher the CNI value, the more deprived the neighbourhood. A high CNI score, i.e. a neighbourhood’s low socioeconomic status, has been considered a risk factor of morbidity and mortality in Swedish data [18].

The sick-leave rate for persons aged 60–64 comprises the number of days receiving sickness benefit, employment injury benefit, rehabilitation benefit and sickness/activities compensation from social insurance in relation to the number aged 60–64 who are registered as insured [19].

Average income represents average municipality figures of cumulative earned income for people ≥65 years of age and encompasses all taxable income except capital gain.

#### Eldercare workload: Registered elderly, residential care facilities places and patients ≥75 years with ≥10 medications

On the primary care level, the proportion of registered older patients together with social deprivation scores is one of the strongest positive predictors of morbidity burden and thus care utilisation [20]. The eldercare workload factors in this study comprise the proportion of registered patients aged ≥65 and ≥80 years per health centre as well as the number of residential care facilities for the elderly places (RCFE places) that the specific health centre is responsible for. We also looked at the proportion of persons aged ≥75 using more than ten medications. In ageing populations, multi-morbidity is associated with taking multiple medications or polypharmacy [21]. All medications in the current medications list (tablets, patches, drops, ointments, injections) at the time of data extraction from the region’s Monitoring Portal (October 2015) were taken into account.

#### Questionnaire

To establish whether PHCCs had a formulated strategy to care for older patients, we developed a questionnaire with the help of Google Web Survey. The online questionnaire contained two questions, which could be answered “Yes” or “No”.

- Do you have any special strategy to avoid hospital readmissions within 30 days for your older patients?
- Do you have a nurse or/and a doctor who is responsible for older patients at your health centre?

### Study procedure

Data for hospital readmission≤30 days for patients aged ≥65 for all PHCCs in RÖC were obtained in August 2015 from the Swedish Quality Portal [22]. CNI, with its parameters, was obtained from Statistics Sweden’s CNI-file report [15]. PHCC-related variables, such as the total number of registered patients, number of registered elderly patients and number of patients aged ≥75 taking ≥10 medications, were obtained from the region’s Monitoring Portal. Data for sick-leave rates and average income of the population in the geographical areas where the PHCCs are located were collected from the Swedish Social Insurance Agency and from Statistics Sweden, respectively.

During September 2015, an e-mail was sent to PHCC managers enquiring how many RCFE places their PHCC was responsible for. In October 2015, the online questionnaire was e-mailed to PHCC managers. Those who did not reply to the online questionnaire received a reminder during January 2016, and those who did not respond to the reminder received a telephone call during March 2016 and they were permitted to answer the same questionnaire questions via telephone. During the telephone interview, no further information was given other than that contained in the e-mail.

### Statistical analyses

Continuous variables are described with the mean, minimum and maximum in the text, and the median has been included in the tables. Categorical variables are described with frequencies or percentages. The correlation between hospital readmission, eldercare workload and socioeconomic conditions is described and analysed with Spearman's correlation coefficient. The significant variables from the correlation analyses were included in a forward stepwise linear regression analysis to identify independent predictors for hospital readmission. The strength of the association will be regarded as very weak (*r* = 0–0.19), weak (*r* = 0.2–0.39), moderate (*r* = 0.40–0.59), strong (*r* = 0.6–0.79) or very strong (*r* = 0.8–1) [23]. The Mann–Whitney *U* test was used for comparison of hospital readmission between two groups. All significance tests were two-sided and performed at the significance level 0.05. IBM SPSS Statistics 22 was used for all statistical analyses.

## Results

Data from the region’s 29 PHCCs were included. The questionnaire was initially answered by 19 PHCCs. A further eight PHCCs answered orally. Two PHCC managers could not be reached by e-mail or telephone, and therefore only 27 of 29 PHCCs answered the questionnaire. All PHCCs responded to the e-mail enquiring about how many RCFE places they were responsible for.

**Figure 1.**
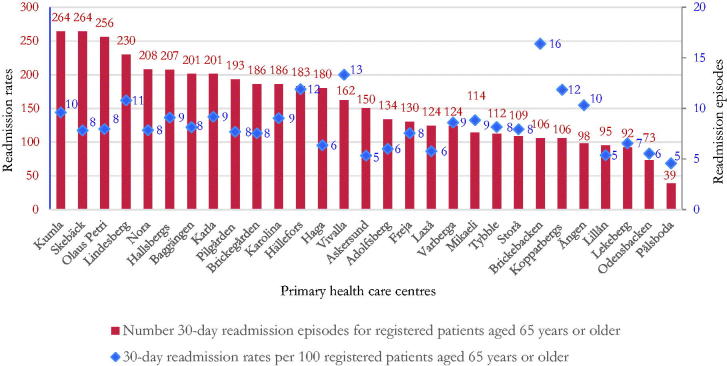
Total number of 30-day hospital readmission episodes (crude) and 30-day readmission rates for patients aged ≥65 in primary health care centres in primary care in Region Örebro County (Sweden).

There were large differences in the number of hospital readmissions per 100 patients ≥65 years across PHCCs, mean (minimum-maximum); 7.7(4.7–14.0) (Figure 1).

Concerning socioeconomic factors, there were large variations across PHCCs in the region, as presented in Table 1.

**Table 1.** Descriptive data for 30-day hospital readmission rates for patients aged ≥65 years, socioeconomic factors, elderly care workload factors and total registered patients for 29 primary healthcare centres in Region Örebro County.

Variable	Mean (Standard Deviation)	Median (Minimum–Maximum)
Number hospital readmissions per 100 registered patients ≥65 years	7.7 (2.1)	7.7 (4.7–14.0)
Total registered patients	9,933.2 (3 868.9)	9,695 (3,202–20,454)
Proportion registered ≥80 years	5.6 (1.7)	5.8 (2.2–8.5)
Proportion registered ≥65 years	21.4 (5.3)	22.5 (10.9–28.5)
Residential care facility for elderly places^†^	88.2 (52.6)	86.0 (10–224)
Proportion patients ≥75 years with ≥10 drugs	7.9 (1.8)	7.3 (5.6–13.3)
Sick-leave days among patients 60–64 years	82.1 (26.6)	79.0 (36–145)
Average income in thousands of SEK for patients ≥65 years	203.0 (31.2)	208.3 (131.3–268.2)
Proportion foreign-born	7.2 (6.4)	5.4 (1.2–33.9)
Proportion unemployed 16–64 years	11.2 (3.2)	10.6 (6.5–22.6)
Proportion low-educated 25–64 years	7.0 (2.1)	7.3 (3.2–13.4)
Care Need Index	1.0 (0.3)	1.0 (0.7–2.0)

† Analysis includes 23 primary healthcare centres that reported residential care facility places for which they were responsible.

### Correlation between hospital readmission, socioeconomics and eldercare workload

The correlation analysis showed strong correlation between hospital readmission and the percentage of persons born outside the EU, EES and North America (*r* = 0.74; *p* < 0.001), the proportion of patients aged 16–64 who were unemployed (*r* = 0.73; *p* < 0.001) and CNI (*r* = 0.74; *p* < 0.001). A moderate correlation (*r* = 0.40–0.59) was observed between hospital readmission and sick-leave rate (*r* = 0.51; *p* < 0.01), and average income (*r* = −0.40; *p* = 0.03). Of the socioeconomic variables studied, the percentage of low-educated patients aged 25–64 did not show a statistically significant relationship to hospital readmission (*r* = 0.27; *p* = 0.16). There were no significant associations between hospital readmission and the proportion of patients aged ≥65 years, the proportion of patients aged ≥80 years, number of RCFE-places, and proportion of patients aged ≥75 years with ≥10 medications. Correlation coefficients are presented in Table 2.

**Table 2.** Spearman’s correlation coefficients between hospital readmission rates and socioeconomic factors and elderly care work load factors.

Variables	Correlation coefficient
Care Need Index	0.74**
Unemployed aged 16–64	0.73**
Foreign-born	0.74**
Low-educated aged 25–64	0.27
Average income for those aged ≥65	−0.40*
Sick-leave rate among patients aged 60–64	0.51**
Registered aged ≥65	−0.25
Registered aged ≥80	−0.11
Registered aged ≥75 with ≥10 drugs	0.30
Residential care facility for elderly places	0.23

* Correlation is significant at the 0.05 level (two-tailed).

** Correlation is significant at the 0.01 level (two-tailed).

**Figure 2.**
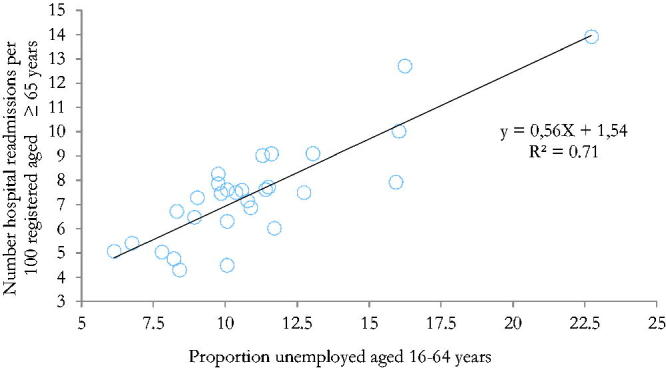
Linear regression between hospital readmission rates and the proportion of unemployed aged 16–64 years. (*Circle-plots represent 29 primary healthcare centres in Region County Örebro*).

A histogram of residuals for hospital readmission showed an approximately normal distribution. All the statistically significant variables from the correlation analysis were entered in the stepwise forward regression analysis. After the proportion of unemployed aged 16–64 years was entered as the strongest variable, the remaining variables were no longer statistically significant. The provided model with regression formula hospital readmission =1.54 + 0.56 × X (X = proportion of unemployed aged 16–64 years) explained 71.4% of the variability in hospital readmission at the individual PHCC (*p* < 0.001), Figure 2.

### Hospital readmission and primary healthcare centre involvement with the older patients

According to the questionnaire, eleven (41%) PHCCs reported that they had a strategy to prevent or reduce the risk for hospital readmission for patients ≥65 years and 16 (59%) had a permanent member of staff, i.e. a nurse or medical doctor, who worked continuously with older patients in their PHCCs or in consultation with the local authorities (Table 3).

**Table 3.** Difference in hospital readmission rates between primary healthcare centres according to whether they reported having strategies for eldercare and staff (designated nurse or doctor) for eldercare.

	*N*	%	Hospital readmission rates			
			Mean (*SD*)	Median (Minimum–Maximum)		*p*-Value for difference
Strategy						
Yes	11	40.7	8.0 (1.4)	7.9 (5.4–10.1)		0.14
No	16	59.3	7.2 (2.0)	7.2 (4.7–12.8)		
Staff						
Yes	16	59.3	7.8 (1.3)		7.9 (5.1–10.2)	0.17
No	11	40.7	7.1 (2.3)		7.4 (4.7–12.8)	

*N* = Number of primary healthcare centres.

*p*-value by Mann–Whitney *U* test.

In the comparison of two groups of PHCCs (Yes vs. No on questionnaire), the Mann–Whitney *U* test showed that PHCCs that reported a strategy against hospital readmission and/or with a nurse or a doctor working specifically with the aged patients did not differ significantly in hospital readmissions from PHCCs without these factors: 8.0 (5.4–10.1) vs. 7.2 (4.7–12.8), *p* = 0.14, respective 7.8 (5.1–10.2) vs. 7.4 (4.7–12.8), *p* = 0.17 (Table 3).

## Discussion

### Main findings

During 2014, there was a large variation in hospital readmission ≤30 days after discharge for patients aged ≥65 between PHCCs in RÖC. There also seemed to be a strong correlation between hospital readmission and socioeconomic conditions among patients at the respective PHCCs: PHCCs located in socioeconomically disadvantaged areas had higher hospital readmission rates for patients aged ≥65 than those in affluent areas. The proportion of unemployed registered persons aged 16–64 years in the area alone could explain up to 71% of the variability in hospital readmission. According to the simple questionnaire we conducted, PHCCs that lacked an active strategy for working with aged patients did not have higher hospital readmission than those that reported having strategies.

### Comments on methods

One limitation with this study is that it only covers one relatively small region of Sweden, with only 29 PHCCs, and it is therefore difficult to comment on whether results can be applied in a larger context. The small sample size could also contribute to the lack of correlation between hospital readmission and some of the other variables. For instance, the correlation between hospital readmission and low-educated inhabitants aged 25–64 years and polypharmacy was not statistically significant in our study, but is still in the same range as was reported in another Swedish study [24].

Another limitation is that data for multi-morbidity, a known predictor for patient readmission, was not available for the current period. Instead, we had to use polypharmacy as a proxy.

Although the information we obtained may lack in depth details because the questionnaire was designed to be simplistic to maximise the possibility to obtain response, the high response rate, 93%, was a strength of our study.

### Comments on results

In this study on Swedish material, we have demonstrated that PHCCs’ figures for early readmission rates for older adults are strongly associated with the socioeconomic characteristics in the area where the PHCC is located. This is in agreement with one other Swedish report and other international studies [24–27], which strengthens our results.

For example, the correlation coefficient for sick-leave rate and average income was in our study comparable to what Larsen reported in 2015. They found sick-leave rate and average income to be strongly correlated with hospital readmission (*r* = 0.50, respectively *r* = −0.46; *p* < 0.001). The age category ≥65 years and socioeconomics could explain up to 60% of the change in hospital readmission. In a qualitative part of their study, where staff at some selected PHCCs were interviewed, no correlation could be found between the reported ambition of the PHCCs to improve quality of care for the frail elderly and the level of hospital readmissions, which also is in accordance with our results [24].

Ageing is associated with multi-morbidity, polypharmacy and healthcare use [6, 28]. According to an ecological study of all general practices in England, social deprivation scores and the proportion of elderly patients were the strongest predictors of morbidity burden and care utilization [20]. In our study, however, we could not find a significant association between the proportion of patients aged ≥65 years, or the proportion of older patients taking more than ten medications (a proxy for multi-morbidity), and hospital readmission. The reason for this lack of association is probably the small sample size. Similar findings were reported by Larsen who included Adjusted Clinical Groups Case-mix system (ACG), a measure of multi-morbidity, and medication review in the analysis [24]. Nevertheless, the literature indicates that healthcare facilities that serve patients in socioeconomically deprived areas tend to have a higher rate of hospital readmission for older adults than those in affluent areas [25]. Some researchers even consider the causes of hospital readmission to be more reflective of socioeconomics and segregation than the quality of care that patients receive and they recommend that hospital readmission should be adjusted for socioeconomics when the variable is used as a quality indicator for comparing healthcare facilities [25, 27].

In our study, the correlations between hospital readmission and those born outside the EU and Europe (*r* = 0.74, *p* < 0.001), those unemployed aged 16–64 (*r* = 0.73, *p* < 0.001) and the CNI (*r* = 0.74, 0.001) were even stronger than in Larsen’s study (*r* = 0.19, 0.40 and 0.50 respectively, *p* < 0.001) [24].

The finding of a strong correlation between hospital readmission rates and socioeconomic factors in our study, especially if this is true for the proportion of foreign-born, might be partly explained by low health literacy. Poor health literacy has been reported in immigrants in Sweden [29] and is associated with increased hospital readmission ≤30 days, elderly mortality and disparities in health outcomes [30]. Also, upon arrival to Sweden, immigrants tend to dissolve strong traditional family bonds, which may result in higher dependence on health care [31].

The causes for early hospital readmission for older patients ≥65 years are multifaceted and complex and seem mostly to depend on factors on individual, organisational and societal levels and to a much lesser extent on how healthcare is shaped on the PHCC level [32]. For example, distance to hospital and socioeconomic status were reported to influence secondary health care use among the adult population in a Swedish county to a large extent [33]. In a Danish study, Heltberg et al. found that although equality in the delivery of diabetes care (pharmacotherapy) was ensured, socioeconomic factors negatively influence attainment of diabetes care goals [34]. Thus, when reports claim that there is a simple connection between quality of healthcare and the number of hospital readmissions, there is every reason to be careful about what conclusions are drawn, because there is always a risk that individual PHCCs and local authorities will have to shoulder the blame unfairly. However, there are examples of successful interventions aiming to reduce hospital readmission, which also could reduce healthcare costs and improve life quality for the older patients [26]. For example, the Kaiser Permanente Readmission Reduction Programme supports the sociodemographic determinants of elderly hospital readmission. It has focused on social aspects of care, moving from disease-specific to patient-focused approach of transition of care with marked reductions in hospital readmissions [35]. Tailored health-promoting programmes in primary care seem capable of achieving health-style improvements in socioeconomically vulnerable people [36].

## Conclusions

In this study, we could not find any correlation between PHCCs that reported a lack of routines for the care of the elderly and hospital readmissions. However, PHCCs in areas with poorer socioeconomic conditions had higher frequencies of hospital readmission among older patients. Further research is needed to clarify what factors are crucial to explain the sociodemographic disparities in early hospital readmissions among people aged ≥65 years, and to find adequate methods for increasing health awareness amongst the most socially vulnerable groups.

## Abbreviations

PHCC: Primary health care centre
RÖC: Region Örebro County
SALAR: Swedish Association of Local Authorities and Regions
SNBHW: Swedish National Board of Health and Welfare
EU: European Union
EES: European Economic Space
CNI: Care Need Index
RCFE places: Residential Care Facilities for Elderly places
